# Mechanical Non-ST-Segment Elevation Myocardial Infarction Secondary to Left Ventricular Outflow Tract Pseudoaneurysm: A Unique Entity

**DOI:** 10.1016/j.jaccas.2022.06.008

**Published:** 2022-08-03

**Authors:** Jathinder Kumar, Rajesh Kumar, Peter Wheen, Ian Pearson, Caroline Daly, Ross Murphy

**Affiliations:** Department of Cardiology, St. James’s Hospital, Dublin, Ireland

**Keywords:** left ventricular outflow tract, non-ST-segment elevation, pseudoaneurysm, ACS, acute coronary syndrome, AVR, aortic valve replacement, CT, computed tomography, LAD, left anterior descending, LCX, left circumflex, LV, left ventricle, LVOT, left ventricular outflow tract, MRI, magnetic resonance imaging, RCA, right coronary artery, TEE, transesophageal echocardiogram

## Abstract

We present a unique case of acute coronary syndrome (ACS) secondary to external coronary artery compression from a left ventricular outflow tract pseudoaneurysm in a postsurgical aortic valve replacement (AVR) patient, subsequently sealed with a pericardial patch. We highlight this rare presentation of ACS in postsurgical AVR patients and the importance of multimodality imaging and treatment of this unique, potentially serious sequela. (**Level of Difficulty: Intermediate.**)

## Central Illustration

**Figure:**
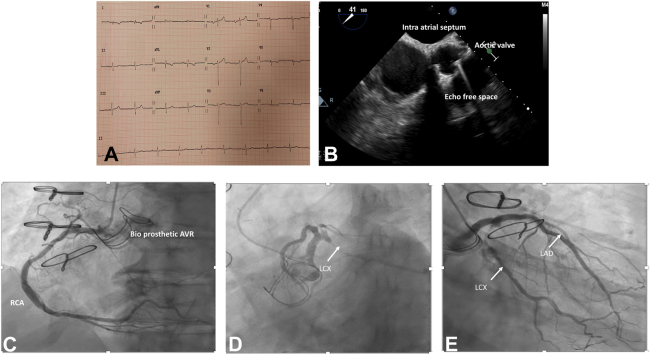

Surgical aortic valve replacement (AVR) remains the gold standard treatment for structural and infective aortic valve diseases. Serious complications after AVR, although rare, include infection, conduction abnormalities, thrombosis, dehiscence, fistula formation, and left ventricular outflow tract (LVOT) pseudoaneurysms.^1^ LVOT pseudoaneurysm is a rare serious complication after cardiac surgery with various clinical presentations, high morbidity, and high mortality.^2^ Various imaging modalities assist in the diagnosis of this complication, such as echocardiography, cardiac gated computed tomography (CT) and magnetic resonance imaging (MRI). This unique case highlights acute coronary syndrome (ACS) secondary to external compression of the coronary artery by a LVOT pseudoaneurysm, its diagnosis, and the importance of imaging in post-AVR patients.

**Learning Objectives**

• To be able to identify unique, almost pathognomonic coronary angiogram findings.
• To be able to recognize late and rare postsurgical repair complications.
• To consider possible left ventricular outflow tract involvement during surgery in the differential diagnosis of infective endocarditis after aortic valve replacement in the absence of valve findings.

## History of Presentation

**Figure 1:**
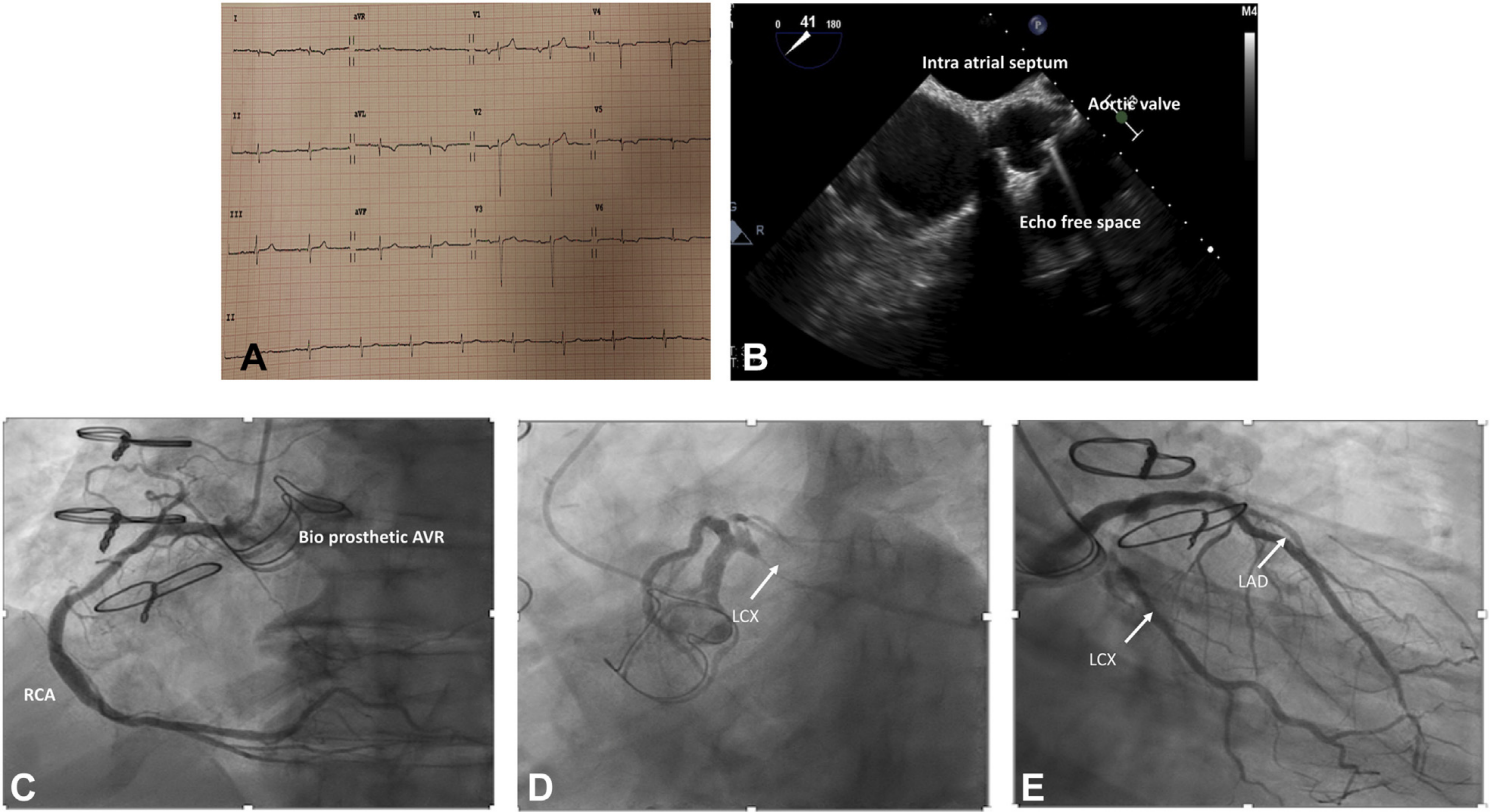
Images of Coronary Arteries **(A)** Electrocardiogram with inferolateral ST-segment depression. **(B)** Transesophageal echocardiogram showing echo-free space. **(C)** Right coronary artery (RCA) showing bioprosthetic aortic valve replacement (AVR). **(D, E)** Left anterior descending (LAD), systolic compression of left circumflex (LCX).

A 69-year-old man was admitted with a 2-day history of sudden-onset, dull central chest pain, 3/10 in severity, with no radiation or associated symptoms. He did not describe any systemic symptoms or fever. His vital signs were within normal range. On examination, he had a normal first heart sound and a prosthetic second heart sound with soft grade 2 systolic murmur. His complete blood count, renal and liver profile, and C-reactive protein were normal. An electrocardiogram (ECG) showed sinus rhythm with inferolateral ST-segment depression (Figure 1A). His serial high-sensitivity cardiac troponin I levels were 239 ng/L→399 ng/L→422 ng/L, respectively (normal, 0-14 ng/L).

## Medical History

His medical history included placement of a surgical bioprosthetic AVR for infective endocarditis 11 months earlier, paroxysmal atrial fibrillation, diabetes mellitus, and coronary artery disease, with percutaneous coronary intervention (PCI) of the right coronary artery (RCA). He was readmitted 3 months after placement of the AVR with stroke, complicated by septicemia. MRI brain confirmed bilateral multiple cerebral and cerebellar infarcts raising the suspicion of septic emboli secondary to possibility of a cardio-embolic phenomenon. Serial blood cultures were negative. A transthoracic echocardiogram showed well seated, functioning prosthetic aortic valve with no evidence of infective endocarditis. A subsequent transesophageal echocardiogram (TEE) confirmed similar findings. He required prolonged hospital admission, treatment with antibiotics for septicemia and extensive rehabilitation.

## Differential diagnosis

Given his history with previous PCI, serial high-sensitivity cardiac troponin I levels, and ECG changes with normal inflammatory markers, type 1 myocardial infarction was considered. The previous surgical AVR raised the possibility of late surgical complications, including potential embolic myocardial infarction, but were thought to be less likely.

## Investigations

**Figure 2:**
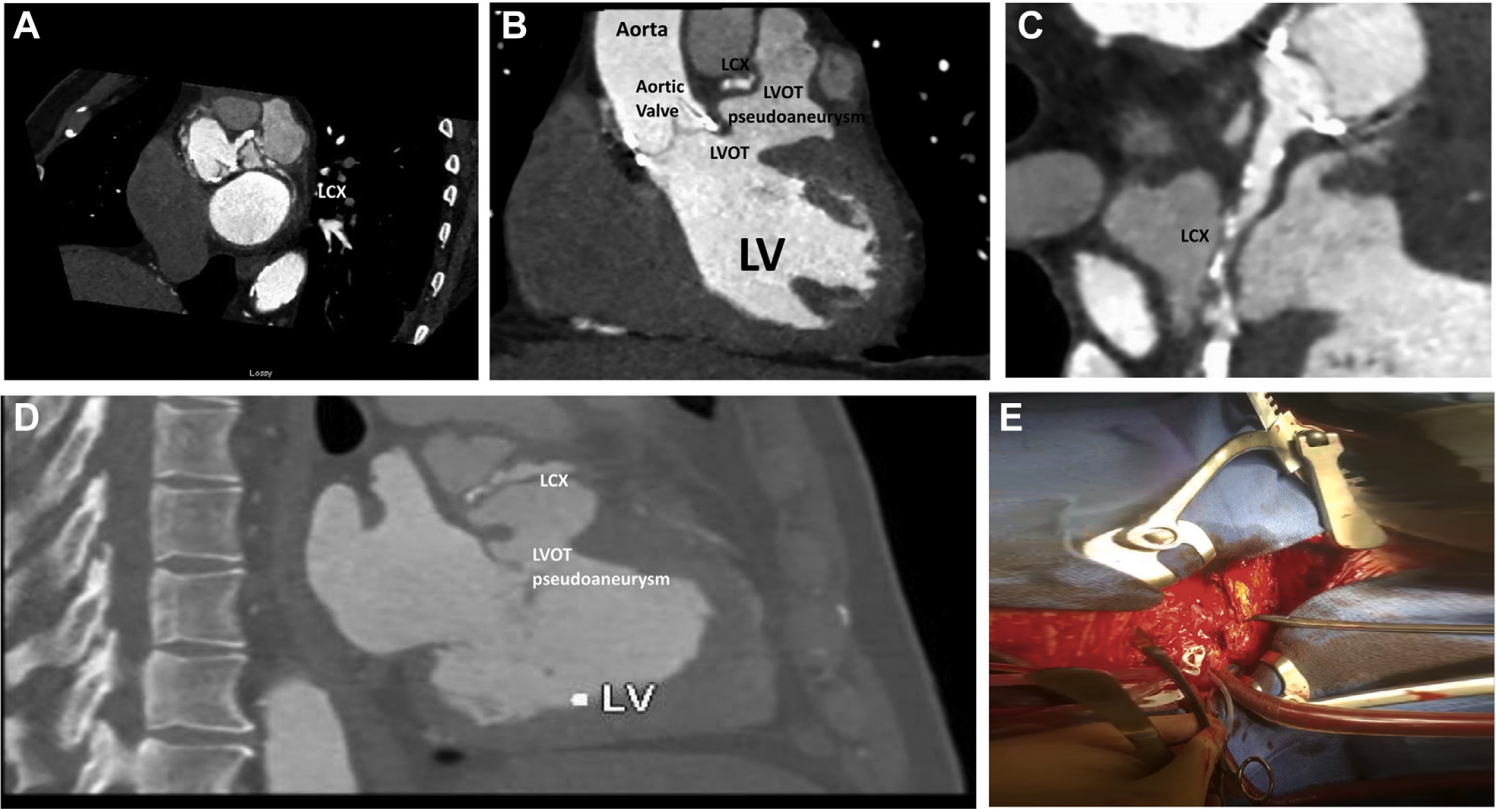
CT and Intraoperative Images of LVOT Pseudoaneurysm **(A to D)** Computed tomography (CT) of aorta/coronary showing left circumflex (LCX) compression by left ventricular outflow tract (LVOT) pseudoaneurysm. **(E)** Bleeding into free space from LVOT pseudoaneurysm.

A transthoracic echocardiogram confirmed moderately impaired left ventricular (LV) ejection fraction of 40%, a well-seated functioning aortic valve prosthesis with no paravalvular leak, vegetations, or abscess, but paravalvular echo-free space. The TEE confirmed paravalvular echo-free space (Figure 1B), AVR with no late features of infective endocarditis. Coronary angiography confirmed nonobstructive RCA with a patent stent (Figure 1C) and mild atheroma in the left anterior descending (LAD) artery. However, prominent systolic external compression of the proximal left circumflex (LCX) artery was observed on fluoroscopy (Figures 1D and 1E), with thrombolysis in myocardial infarction II flow in the distal part of the vessel (Videos 1 and 2). A subsequent computed tomography (CT) angiogram of the coronary vessels and aorta confirmed an 8-mm fistula arising from the LVOT, inferior to the aortic annulus, filling an irregular space. The free space, approximately 6 cm in diameter, was lateral to the LAD and the main pulmonary artery, draining freely into the pericardium, which was contained, leading to external compression of the LCX, explaining the patient’s ACS presentation (Figures 2A to 2D).

## Management

The patient underwent a redo sternotomy with repair of the pseudoaneurysm after a heart team discussion. The intraoperative surgical findings correlated with the CT findings. The pseudoaneurysm (Figure 2E, Video 3) was deroofed, and the LVOT defect (1 cm) was closed directly by the use of 3.0 polypropylene sutures with pericardial pledgets. The aortic valve prosthesis was normal, with no vegetations or abscess. The patient did not require coronary artery bypass grafting. The deroofed tissue was cultured and did not yield any growth after 5 days. The patient’s postsurgical recovery was uncomplicated, and he was discharged home in stable condition, receiving optimal medical treatment for LV dysfunction.

## Follow-Up

The patient was in clinically stable condition after surgery and received follow-up care in the heart support unit with optimization of medical treatment for moderate LV dysfunction.

## Discussion

Ventricular pseudoaneurysm is a contained cardiac rupture encircled by adherent pericardium or scar tissue, with no myocardial tissue. An LVOT pseudoaneurysm after AVR is an uncommon but serious complication. The predisposing factors include infective endocarditis, prosthetic valve degeneration, or suture dehiscence after valvular surgery.^1^ It commonly occurs after myocardial infarction or after cardiac surgery, as was seen in this case. Pseudoaneurysms are unstable, given that they are contained. It is important to differentiate pseudoaneurysms from true aneurysms because they are managed differently. Pseudoaneurysms can lead to life-threatening complications, such as compression of local structures and vasculature secondary to mass effect. Pseudoaneurysms are prone to spontaneous thrombosis, or rupture because of their enlarging size and embolic phenomenon secondary to turbulent or slow flow.^3^^,^^4^ In noninfective cases, the formation of a pseudoaneurysm is usually related to larger aortic root diameter and changes in the aortic annulus after AVR.^5^

LVOT pseudoaneurysms have various clinical presentations ranging from the patient’s being asymptomatic to critically ill. They can present with generalized systemic symptoms, symptoms of an infective process, embolic events, and sometimes anginal symptoms that can mimic ACS or coronary artery disease, secondary to compression of the coronary vasculature as described by Schaap et al.^4^

Various imaging modalities are used in the diagnosis of LVOT pseudoaneurysms such as echocardiography, ECG-gated cardiac CT, and cardiac MRI. Barbetseas et al^6^ used Doppler echocardiography to evaluate pseudoaneurysm formation after AVR and suggested that such techniques can diagnose pseudoaneurysms complicating composite aortic grafts. ECG-gated cardiac CT is a useful tool in the assessment of prosthetic valve complications after surgery and infective endocarditis and can also guide surgical correction.^3^ Cardiac MR, the gold standard, is highly valuable in differentiating pseudoaneurysms from true aneurysms,^7^ pericardium from myocardium, scar or hematoma, and in identifying the location of the aneurysm.

The treatment of pseudoaneurysms after AVR depends on their anatomical location, size, and type. The treatment options include surgical closure, or percutaneous closure for patients at high risk for surgical closure because of comorbidities. Pseudoaneurysms left untreated or undiagnosed have a high risk of spontaneous rupture secondary to their thinner walls and higher-flow dynamics. Surgical repair, however, carries a high risk of morbidity and mortality—approximately 20% to 36%^8^—but still retains a better prognosis than does conservative management.^8^

Percutaneous closure of a LV pseudoaneurysms with a closure device is an option in patients at prohibitively surgical risk, but it can be challenging because there is no standardized approach to, and fluoroscopic views for, closure of LV pseudoaneurysms. The percutaneous options are technically difficult and require multiple imaging modalities. The use of 3-dimensional echocardiography and CT angiography can be helpful in these cases.^9^ Device dislodgement and embolization constitute the rare complication associated with percutaneous closure.

Another important consideration is the need for rescue coronary artery bypass grafting resulting from compromised coronary circulation as a result of the compressive disease process as described by Shahriari et al.^10^

Our patient presented with non-ST-segment elevation myocardial infarction resulting from of proximal LCX external compression from an LVOT pseudoaneurysm. TEE confirmed paravalvular echo-free space, and coronary angiography showed very unusual stenosis of the proximal LCX with intermittent compromised flow in the mid-distal segment of the vessel during systole. Finally, CT angiogram of the coronary vessels and the aorta confirmed LVOT pseudoaneurysm, which was successfully treated by redo sternotomy and surgical deroofing. This unique case highlights the importance of multi-imaging modality in the diagnosis of ACS after cardiac surgery, its complications, and various treatment options based on anatomical findings.

## Conclusions

Compression of the coronary vasculature secondary to pseudoaneurysm after cardiac surgery is a rare life-threatening complication with a unique classic angiographic appearance. Multimodality imaging is needed to confirm the diagnosis and to tailor management accordingly. Awareness of this entity among cardiologists will enable early diagnosis and timely management of this condition, with potential prognostic benefit.

## Funding Support and Author Disclosures

The authors have reported that they have no relationships relevant to the contents of this paper to disclose.

## Appendix

Supplemental Video 1 Coronary angiography showing compression of left circumflex artery

Supplemental Video 2 Coronary angiography showing compression of left circumflex artery

Supplemental Video 3 Intraoperative bleeding into free space from pseudoaneurysm of left ventricular outflow tract
